# SYT8 promotes pancreatic cancer progression via the TNNI2/ERRα/SIRT1 signaling pathway

**DOI:** 10.1038/s41420-021-00779-4

**Published:** 2021-12-14

**Authors:** Zhiping Fu, Xing Liang, Ligang Shi, Liang Tang, Danlei Chen, Anan Liu, Chenghao Shao

**Affiliations:** Department of Pancreatic-Biliary Surgery, Second Affiliated Hospital of Naval Medical University, Shanghai, China

**Keywords:** Protein-protein interaction networks, Pancreatic cancer

## Abstract

Pancreatic cancer is a highly lethal malignancy due to failures of early detection and high metastasis in patients. While certain genetic mutations in tumors are associated with severity, the molecular mechanisms responsible for cancer progression are still poorly understood. Synaptotagmin-8 (SYT8) is a membrane protein that regulates hormone secretion and neurotransmission, and its expression is positively regulated by the promoter of the insulin gene in pancreatic islet cells. In this study, we identified a previously unknown role of SYT8 in altering tumor characteristics in pancreatic cancer. SYT8 levels were upregulated in patient tumors and contributed towards increased cell proliferation, migration, and invasion in vitro and in vivo. Increased SYT8 expression also promoted tumor metastasis in an in vivo tumor metastasis model. Furthermore, we showed that SYT8-mediated increase in tumorigenicity was regulated by SIRT1, a protein deacetylase previously known to alter cell metabolism in pancreatic lesions. SIRT1 expression was altered by orphan nuclear receptor ERRα and troponin-1 (TNNI2), resulting in cell proliferation and migration in an SYT8-dependent manner. Together, we identified SYT8 to be a central regulator of tumor progression involving signaling via the SIRT1, ERRα, and TNNI2 axis. This knowledge may provide the basis for the development of therapeutic strategies to restrict tumor metastasis in pancreatic cancer.

## Introduction

Pancreatic cancer is one of the leading causes of cancer-related deaths worldwide with a 5-year survival rate as low as 5% from the time of diagnosis [1]. The high mortality rate is largely due to the limited availability of effective prognostic markers that would allow for early detection, as well as the highly invasive nature of pancreatic cancer, which leads to metastasis to distant organs [2]. The risk factors which predispose individuals to pancreatic cancer include age and smoking, while gene mutations are one of the key causes of pancreatic cancer and its metastasis [3, 4]. The most commonly known pancreatic cancer-associated oncogene is KRAS, which is activated in most pancreatic tumors [4]. Activated Ras further triggers the RAF/mitogen-activated protein and phosphoinositide-3-kinase/AKT signaling pathways that activate key downstream transcription factors to promote cell proliferation [5]. Other commonly known mutations are observed in CDKNA, TP53, and SMAD4 tumor-suppressor genes, which inactivate gene functions and promote aberrant cancer progression [6–8].

Aberrant carcinoma of the pancreas has been shown to lead to deficient or uncontrolled hormone production, including insulin that regulates blood sugar levels [9]. Synaptotagmin-8 (SYT8) is a membrane protein that actively plays a role in exocytosis and vesicle secretion [10, 11] and has been implicated in insulin secretion [12]. Its neighboring troponin I (*TNNI2)* gene is an inhibitory subunit of troponin that regulates calcium-dependent muscle ATPase activity [13]. While the roles of SYT8 and TNNI2 have not been broadly described in the context of cancer, their expression levels have been shown to be increased in gastric and bladder-related cancers, indicating that SYT8 and TNNI2 could act as prognostic markers [14–16]. Although SYT8 can reduce insulin secretion in a Ca^2+^-dependent manner in islet cells, it does not act as an active calcium sensor in other cell types [10, 11]. Xu et al. reported that *SYT8* expression, along with *TNNI2*, can be induced by the insulin promoter by long-range interactions via altered chromatin architecture. Increased insulin production or hyperinsulinemia has been largely associated with pancreatic cancer progression [17], but the molecular mechanisms associated with it have not been clearly elucidated.

Sirtuin-1 (SIRT1) is a NAD-dependent protein deacetylase that regulates pathways related to metabolism and autophagy [18]. High SIRT1 expression has been associated with poorly differentiated pancreatic ductal carcinomas and poor disease outcomes [19]. In addition, SIRT1 has been shown to promote the proliferation and metastasis of human pancreatic cancer cells [20]. Interestingly, SIRT1 acts as a key metabolic regulator in pancreatic cells, to promote the expression of glycolytic genes that contribute to the development of neoplastic lesions [21]. Thus, SIRT1 is an important regulatory protein in pancreatic cancer.

Estrogen-related receptor alpha (ERRα) is a nuclear receptor (NR) with high sequence similarity to the estrogen receptor, but with a poor affinity towards estrogen binding, and is widely expressed in metabolically active cells [22, 23]. It has diverse roles such as in cardiac maturation and regulation of mitochondrial biogenesis [24, 25]. In the context of cancer, ERRα positively regulates growth in oral squamous cell carcinoma, bladder cancer, and prostate cancer [26–28]. SIRT1 has not only been shown to act as a coactivator of ERRα transcriptional activity but ERRα has also been found to transcriptionally activate SIRT1 in macrophages [29, 30]. Furthermore, we identified SIRT1 as an interacting component of ERRα using the ToppGene Suite portal (https://toppgene.cchmc.org). However, the functional consequence of this interaction and its role in pancreatic cancer has not yet been determined.

In the present study, we used pancreatic cancer cell lines in vitro, an in vivo tumor mouse model, and pancreatic cancer patient tissue samples to identify a novel SYT8-dependent molecular mechanism mediating pancreatic cancer cell proliferation and invasion via TNNI2, ERRα, and SIRT1. Our study identifies SYT8 as a potential prognostic marker of pancreatic cancer and provides a deeper mechanistic understanding of pancreatic cancer progression.

## Results

### SYT8 is highly expressed in pancreatic cancer tissues

**Fig. 1:**
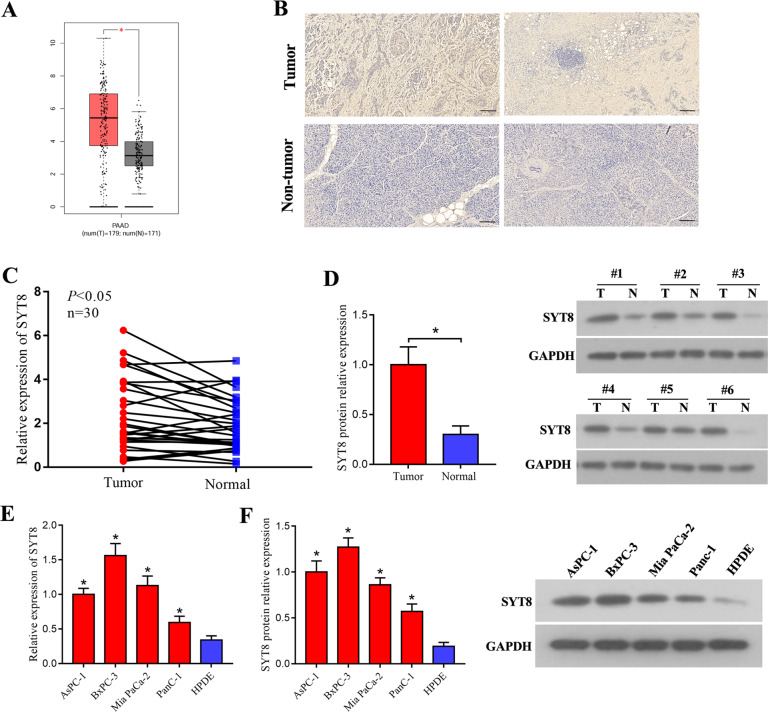
Upregulation of SYT8 expression in pancreatic cancer. **A** The mRNA levels of SYT8 in pancreatic cancer patient tissues (*n* = 179) and pancreatic non-tumor tissues (*n* = 171) using the GEPIA web tool. **B** Representative images from the immunohistochemical analysis showing SYT8 expression in pancreatic cancer tissues and pancreatic non-tumor tissues from two patients with pancreatic cancer. Scale bar: 100 μm. **C** The mRNA expression levels of SYT8 in 30 paired tumor and adjacent non-tumor tissues from pancreatic cancer patients were quantified by qRT-PCR. **D** SYT8 protein expression quantification and representative images from western blotting were performed in pancreatic cancer patient specimens and adjacent non-tumor tissues. **E**, **F** SYT8 mRNA and protein expression levels in pancreatic cell lines. The qRT-PCR analysis, densitometric quantification, and representative images of western blotting from HPDE, AsPC-1, BxPC-3, PANC-1, and MIA PaCa-2 cells. Data are presented as mean ± SD (*n* = 3). *P < 0.05.

To investigate the effects of SYT8 in the context of pancreatic duct adenocarcinoma, we analyzed 179 patient tumor tissues and 171 non-tumor tissues for expression of SYT8 using the Gene Expression Profiling Interactive Analysis (GEPIA) platform. This analysis revealed that SYT8 levels were significantly higher in tumor tissues when compared with non-tumor healthy tissues (Fig. 1A). Immunohistochemical staining of tissue sections also indicated an increase in SYT8 expression when compared to adjacent healthy tissue sections from patients (Fig. 1B). We further quantified mRNA expression levels of SYT8 in 30 pairs of pancreatic tumor and healthy tissues from patients by quantitative reverse transcription polymerase chain reaction (qRT-PCR) and observed a moderate but significant increase in SYT8 expression (Fig. 1C). Protein expression analysis by western blotting showed a twofold increase in SYT8 levels in tumor tissues when compared with healthy samples (Fig. 1D). To verify these findings in various in vitro cell line models, we used four different pancreatic cell lines AsPC-1, BxPC-2, Mia PaCa-2, and PANC-1 and compared them with the normal human pancreatic duct epithelial (HPDE) cell line. All pancreatic cancer cell lines showed a significantly higher increase in SYT8 mRNA and protein expression levels when compared to HPDE cells (Fig. 1E, F). These results showed that SYT8 expression was significantly upregulated in pancreatic cancer.

### SYT8 enhances cell proliferation in vitro and pancreatic tumor growth in vivo

**Fig. 2:**
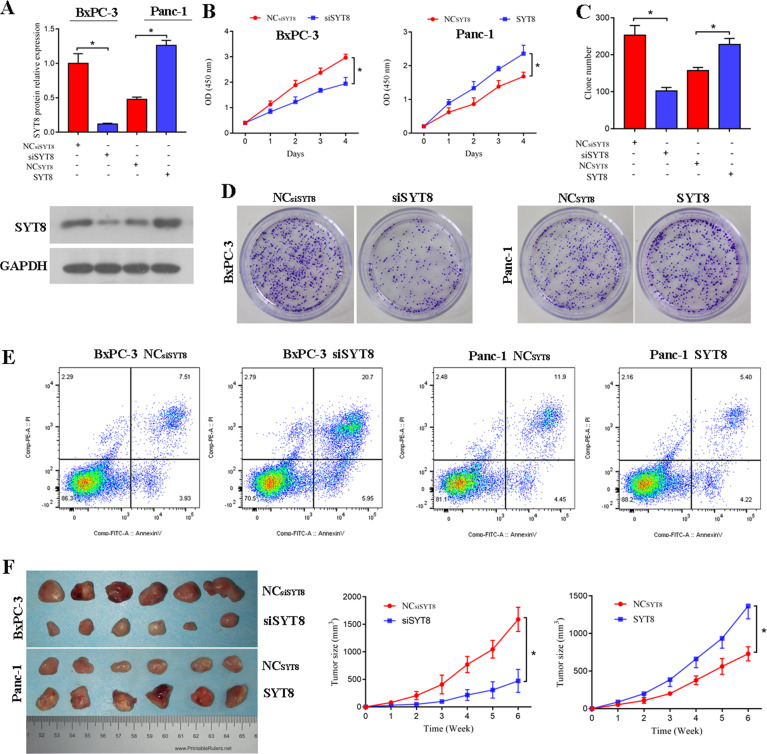
SYT8 promotes cell proliferation in vitro and tumor growth in vivo. **A** SYT8 protein expression levels in BxPC-3 and PANC-1 cells 48 h post-transfection of non-targeting scrambled siRNA (NCsiSYT8) or siRNA SYT8 (siSYT8); the SYT8 overexpression vector or the respective control vector (NCSYT8) was analyzed by western blotting. **B** Cell viability in BxPC-3 and Panc-1 cell lines post-transfection using the CCK-8 assay, with the absorbance measured at 450 nm. **C**, **D** Quantification and representative images from the colony-formation assay of cell viability in BxPC-3 and PANC-1 cell lines. **E** Cell apoptosis. Representative dot plots of SYT8-knockdown BxPC-3 cells and SYT8-overexpressing PANC-1 cells stained for Annexin-V/PI and measured by flow cytometry. **F** In vivo tumor growth assay. BALB/c nude mice were injected with BxPC-3 or PANC-1 cells expressing the respective siRNA or overexpression vector and monitored every week for up to 6 weeks. Tumors were then harvested to measure the volume. *n* = 6 per group. Data are presented as mean ± SD with at least three independent experiments. *P < 0.05.

Because SYT8 has been reported to favor tumor invasion and metastasis in gastric cancer [31], we next sought to determine the role of SYT8 in altering tumor characteristics in pancreatic cancer cell lines. To this end, we used small interfering RNA (siRNA)-mediated silencing of SYT8 (siSYT8) or overexpression of SYT8 in BxPC-3 and PANC-1 cells, respectively. Upon treatment of BxPC-3 cells with siSYT8 for 48 h, we observed a more than threefold decrease in mRNA levels as quantified by qRT-PCR. Furthermore, overexpression of SYT8 led to a more than twofold increase in SYT8 levels in PANC-1 cells (Fig. 2A). Next, we examined the effects of SYT8 knockdown and overexpression on cell viability and proliferation. Knockdown of SYT8 significantly reduced proliferation in BxPC-3 cells, while SYT8 overexpression promoted cell proliferation in PANC-1 cells (Fig. 2B). Similarly, our colony formation assays revealed that knockdown or overexpression of SYT8 significantly inhibited or promoted cell growth, respectively (Fig. 2C, D). We further evaluated the effect of SYT8 on cell apoptosis by Annexin-V/propidium iodide (PI) staining and flow cytometry. BxPC-3 cells expressing siSYT8 showed a higher level of apoptosis as indicated by positive staining for Annexin-V and PI, whereas overexpression of SYT8 reduced this effect (Fig. 2E). Furthermore, when siSYT8-expressing or SYT8-overexpressing BxPC-3 or PANC-1 cells were transferred to BALB/c nude mice and monitored for tumor growth, we observed a significant decrease in tumor size over 6 weeks in the siSYT8-expressing mice compared to an increase in the SYT8 overexpression group (Fig. 2F). Taken together, these results suggested that SYT8 directly regulates cell survival and tumor growth in pancreatic cancer.

### SYT8 contributes to pancreatic cancer cell invasion and metastasis

**Fig. 3:**
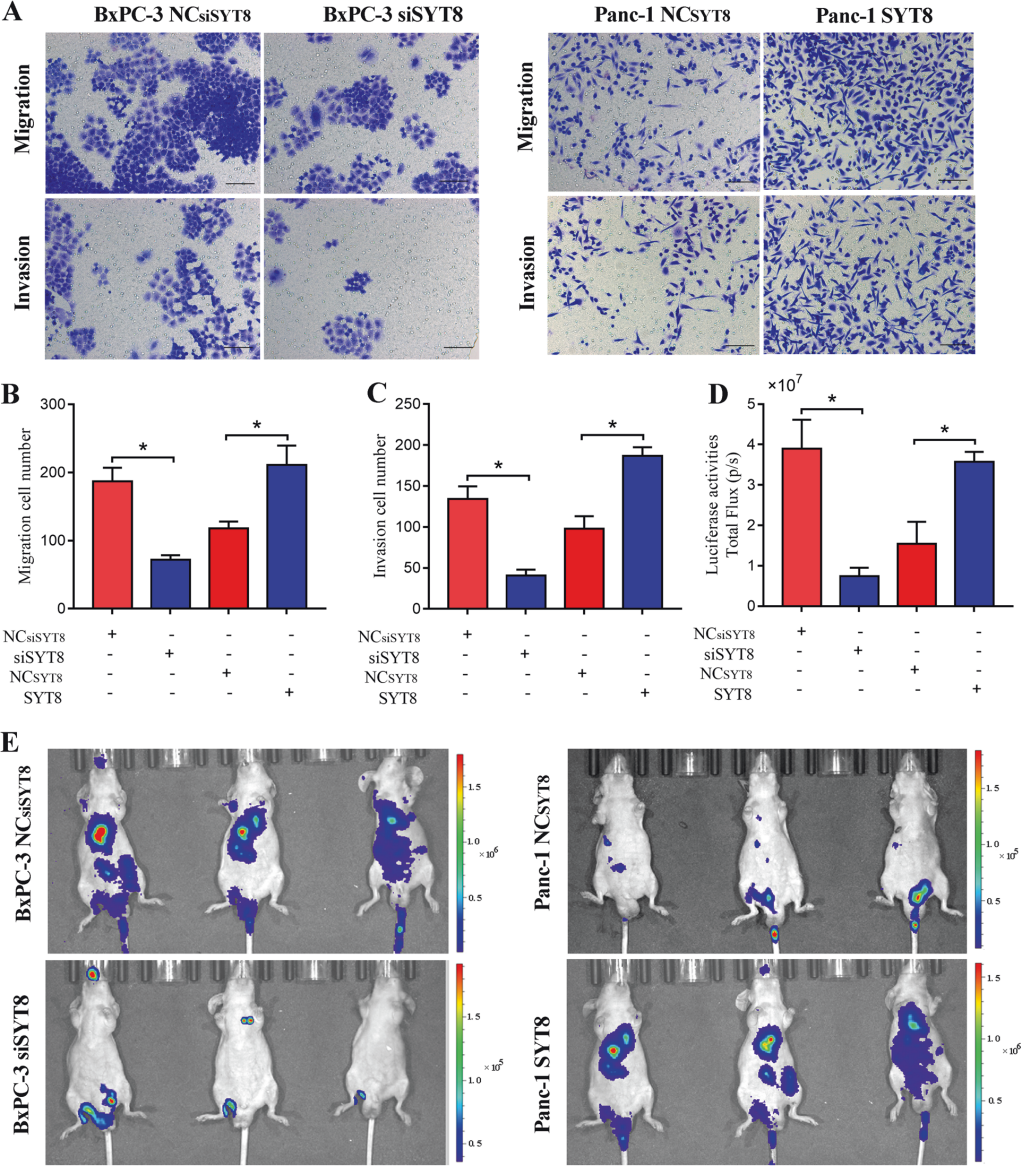
SYT8 promotes metastasis in vitro and in vivo. **A**–**C** Migration and invasion assays. BxPC-3 and PANC-1 cell lines expressing the different vectors were seeded into Transwell chambers. At 48 h post-seeding, the upper chamber was removed, and cells migrating to the lower chamber were visualized by Crystal Violet staining and quantified. Representative images and quantification of absolute numbers of cells migrated or invaded to the lower chamber. Scale bar: 100 μm. **D**, **E** In vivo metastasis assay. BxPC-3NC or BxPC-3siSYT8 and PANC-1NC or PANC-1SYT8 cells tagged with luciferase were transferred into nude BALB/c mice via tail vein injections. Organ metastases were assessed weekly using a bioluminescence imaging system. Quantification of luciferase expression measured as total flux (**D**) and representative scan images (**E**) are shown. *n* = 6 per group. Data are presented as the mean ± SD with at least three independent experiments. *P < 0.05.

To further understand the role of SYT8 in favoring pancreatic cancer, we performed in vitro migration and in vivo imaging analyses. siSYT8-expressing or SYT8-overexpressing BxPC-3 or PANC-1 cells were seeded onto Transwell inserts and migration/invasion was observed after 48 h. Knockdown of SYT8 reduced cell migration and invasion twofold in BxPC-3 cells, while overexpression of SYT8 increased these effects twofold in PANC-1 cells (Fig. 3A–C). Metastasis was assessed by injecting luciferase-tagged BxPC-3 (NC), BxPC-3 (siSYT8), PANC-1 (NC), or PANC-1 (SYT8) cells into the tail vein of mice and imaging weekly to observe metastasis by bioluminescence. As expected, mice that received cells expressing siSYT8 showed significantly lower metastasis quantified as the amount of luciferase units, whereas overexpression of SYT8 significantly promoted metastasis (Fig. 3E, D). In summary, our results indicated that SYT8 plays an important role in both promoting cell invasion in vitro and tumor metastasis in vivo.

### SYT8 promotes pancreatic cancer progression through SIRT1

**Fig. 4:**
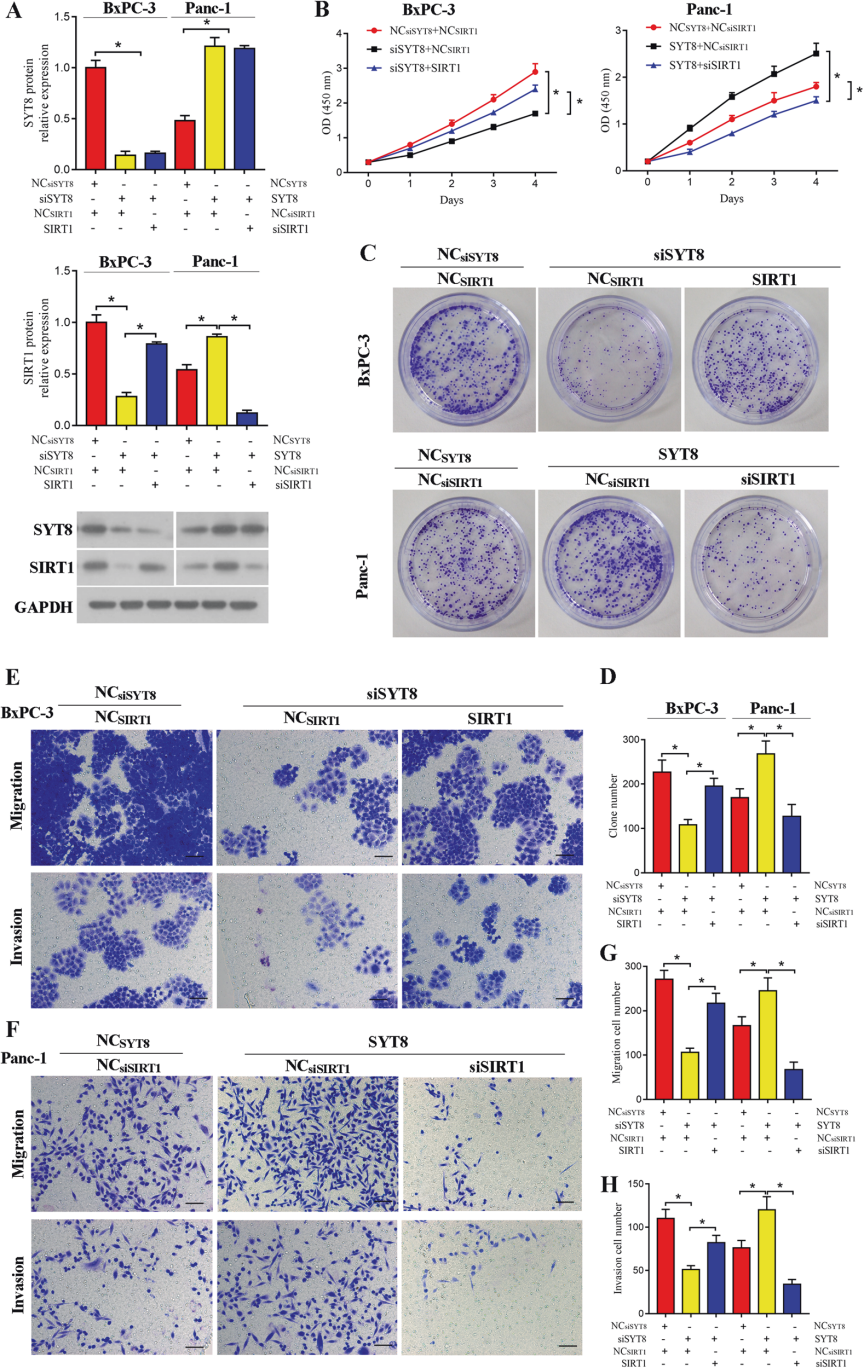
SYT8 enhances proliferation and metastasis of pancreatic cancer cells by upregulating SIRT1. **A** Densitometric quantification and representative western blot images showing SYT8 and SIRT1 protein expression levels in BxPC-3 and PANC-1 cells co-expressing different siRNA or overexpression vectors against SYT8 and SIRT1. Glyceraldehyde 3-phosphate dehydrogenase was used as a loading control. **B** Cell proliferation was measured by CCK-8 analysis in cells expressing the different constructs measured over 4 days post-transfection. **C**, **D** Representative images and quantification of total colonies formed in BxPC-3 or PANC-1 cells expressing different vectors. **E**–**H** Representative images and quantification of cell migration and invasion in BxPC-3 or PANC-1 cells were measured using a Transwell system 48 h post-transfection. Scale bar: 100 μm. Data are presented as mean ± SD with at least three independent experiments. *P < 0.05.

To identify the mechanism behind SYT8-mediated regulation of tumor metastasis, we investigated the role of SIRT1, a protein deacetylase that plays a wide role in regulating the cell cycle during apoptosis [32]. We transfected the SIRT1 overexpression construct into siSYT8-expressing BxPC-3 cells and siSIRT1 into SYT8-overexpressing PANC-1 cells to study the relationship between SYT8 and SIRT1 in pancreatic cancer. Quantifications of mRNA and protein expression levels revealed a robust knockdown or overexpression of SYT8 and SIRT1 in both BxPC-3 and PANC-1 cells (Fig. 4A). Knockdown of SYT8 also decreased levels of SIRT1 expression in BxPC-3 cells (Fig. 4A). Overexpression of SIRT1 significantly improved cell proliferation of siSYT8-expressing cells, while knockdown of SIRT1 reduced cell proliferation of SYT8-overexpressing cells (Fig. 4B). Similar effects were observed in cell growth, migration, and invasion, where overexpression of SIRT1 significantly improved the proliferative, migratory, and invasive abilities of cells expressing siSYT8, and knockdown of SIRT1 significantly alleviated the effects of SYT8 overexpression (Fig. 4C–H). These results showed that SIRT1 exerts a function similar to SYT8 in favoring cell proliferation and migration in pancreatic cancer cell lines.

### SYT8-upregulated SIRT1 expression is dependent on ERRα in pancreatic cancer cells

**Fig. 5:**
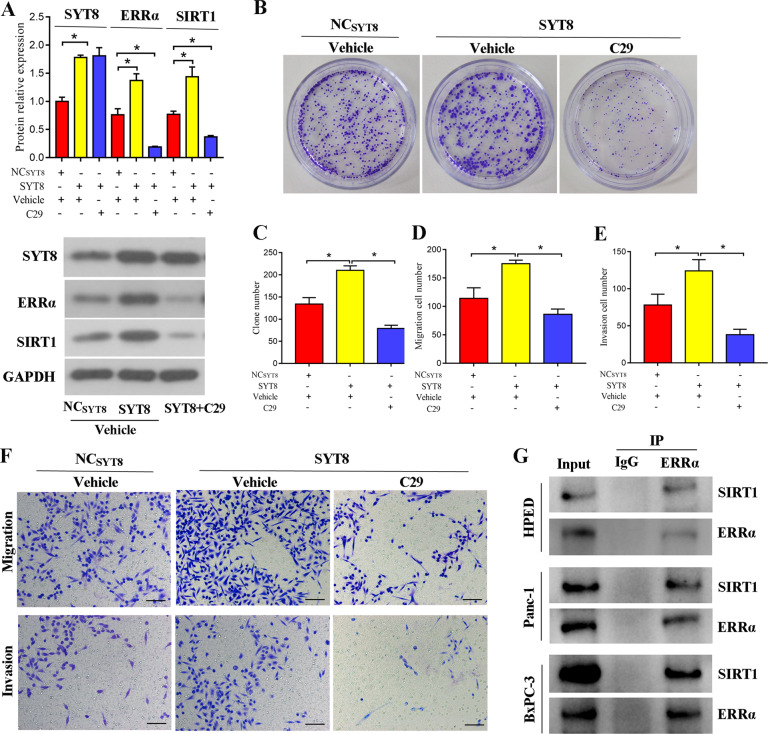
ERRα favors SYT8-induced SIRT1 expression and pancreatic cancer progression. **A** Densitometric quantification and representative western blot images showing SYT8, SIRT1, and ERRα protein expression levels in PANC-1 cells transfected with control (NC) or SYT8 overexpression vectors treated with a vehicle or C29, an inhibitor against ERRα for 24 h. **B**, **C** Representative images and quantification from colony-formation analyses of PANC-1 cells overexpressing SYT8 in the presence and absence of the inhibitor. **D**–**F** Quantification and representative images from Transwell migration and invasion assays. Scale bar: 100 μm. **G** Representative images from western blots. Immunoprecipitates with ERRα-coated beads using cell lysates from HPDE, BxPC-3, and Panc-1 cells were subjected to electrophoresis, blotted onto membranes, and probed with antibodies to visualize SIRT1 and ERRα expression. Data are presented as mean ± SD with at least three independent experiments. *P < 0.05.

To further gain insight into the mechanism by which SYT8 and SIRT1 mediated cancer progression, we investigated the orphan NR, ERRα, which was identified as a SIRT1 interactor by bioinformatics analysis. We used SYT8-overexpressing PANC-1 cells and treated them with C29 (an inhibitor against ERRα) for 24 h and evaluated SYT8, ERRα, and SIRT1 levels. As expected, the inhibitor robustly reduced ERRα expression levels, whereas SYT8 levels remained unchanged (Fig. 5A). Notably, SIRT1 protein levels showed a threefold reduction upon inhibitor treatment in SYT8-overexpressing PANC-1 cells (Fig. 5A). Furthermore, inhibitor treatment also reduced cell proliferation, migration, and invasion of SYT8-overexpressing PANC-1 cells as measured by colony formation and Transwell assays, respectively (Fig. 5B–F). Immunoprecipitation of endogenous ERRα using HPED, PANC-1, and BxPC-3 cell lysates mixed with ERRα-coated beads showed an association between SIRT1 and ERRα (Fig. 5G). Taken together, these results indicated that ERRα was associated with SIRT1 and altered its expression levels, leading to reduced cell proliferation, migration, and invasion in pancreatic cell lines.

### TNNI2 directly activates the ERRα/SIRT1 pathway to promote pancreatic cancer progression

**Fig. 6:**
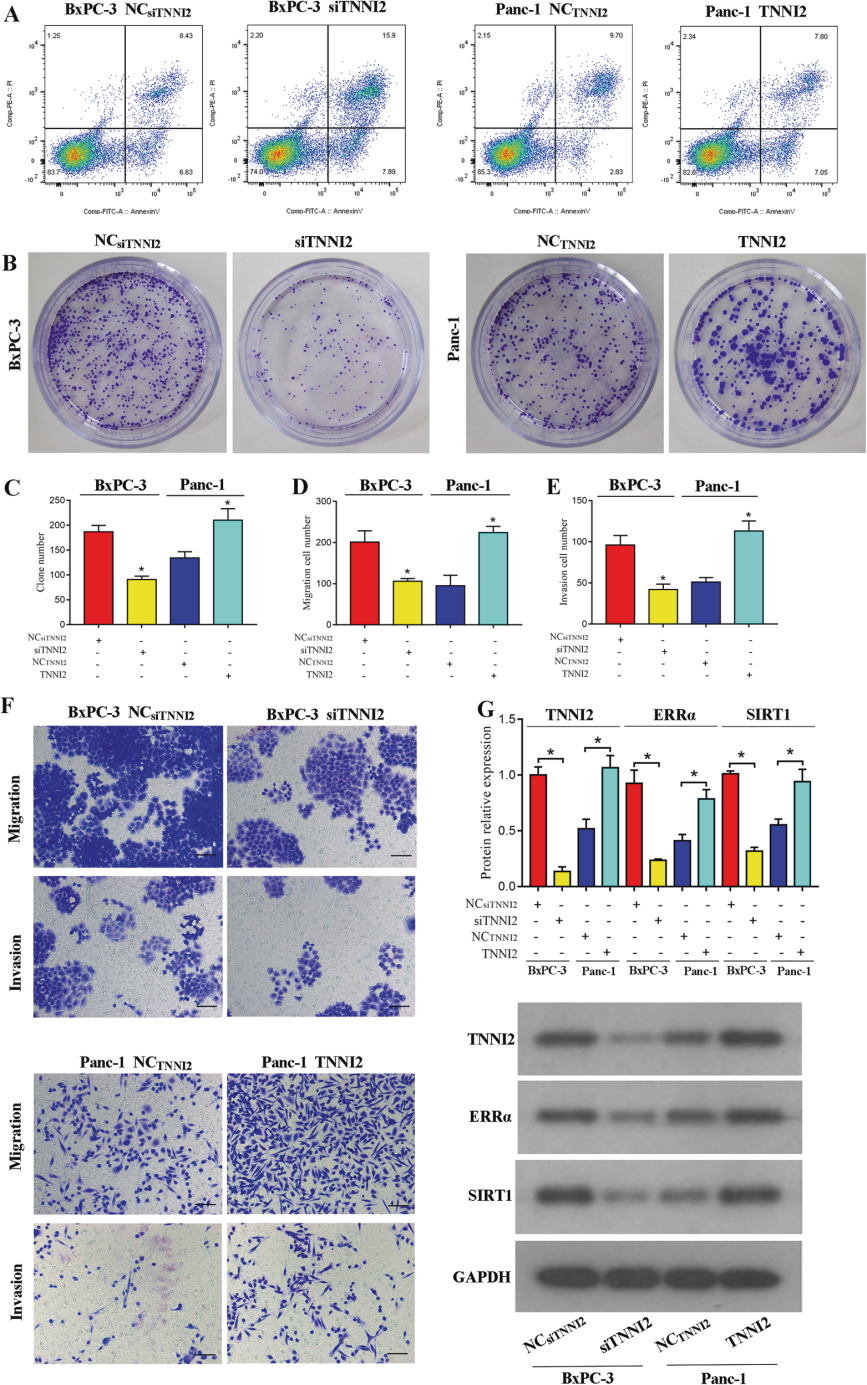
TNNI2 promotes pancreatic cancer progression by directly regulating the ERRα/SIRT1 pathway. **A** Representative dot plots from flow cytometric analysis of apoptosis in the indicated cells measured after Annexin-V/propidium iodide staining. **B**, **C** Representative images and quantification of colony-formation assays in BxPC-3 and Panc-1 cells expressing either siTNNI2 or TNNI2 overexpression vector, respectively. **D**–**F** Representative images and quantification of cell migration and invasion in BxPC-3 or PANC-1 cells were measured using a Transwell system 48 h post-transfection. Scale bar: 100 μm. **G** Densitometric quantification and representative western blot images showing TNNI2, ERRα, and SIRT1 protein expression levels in BxPC-3 and PANC-1 cells expressing either siRNA or the overexpression vector against TNNI2. Data are presented as mean ± SD with at least three independent experiments. *P < 0.05.

TNNI2 is a co-activator and a direct interactor of ERRα [33]. Hence, we evaluated its role in BxPC-3 and PANC-1 cells using siRNA or overexpression constructs against TNNI2. The siRNA knockdown of TNNI2 (siTNNI2) greatly reduced cell proliferation as observed by colony formation assay, and overexpression of TNNI2 promoted this effect (Fig. 6B, C). Evaluation of apoptosis showed an increase in Annexin-V/PI expression in siTNNI2-expressing BxPC-3 cells as a measure of increased apoptosis and a relative decrease in TNNI2-overexpressing PANC-1 cells (Fig. 6A). Similarly, cell migration and invasion were also significantly reduced upon siTNNI2 expression and increased upon overexpression of TNNI2 (Fig. 6D–F). Further analysis of protein expression levels showed that upon TNNI2 knockdown, expression of both ERRα and SIRT1 was significantly reduced in BxPC-3 cells and upon overexpression, these levels were upregulated (Fig. 6G). In summary, these results showed a direct correlation between TNNI2 and the ERRα/SIRT1 signaling pathway and its role in altering cell characteristics in pancreatic cancer cell lines.

### TNNI2 is required for SYT8-induced activation of the ERRα/SIRT1 pathway in pancreatic cancer cells

**Fig. 7:**
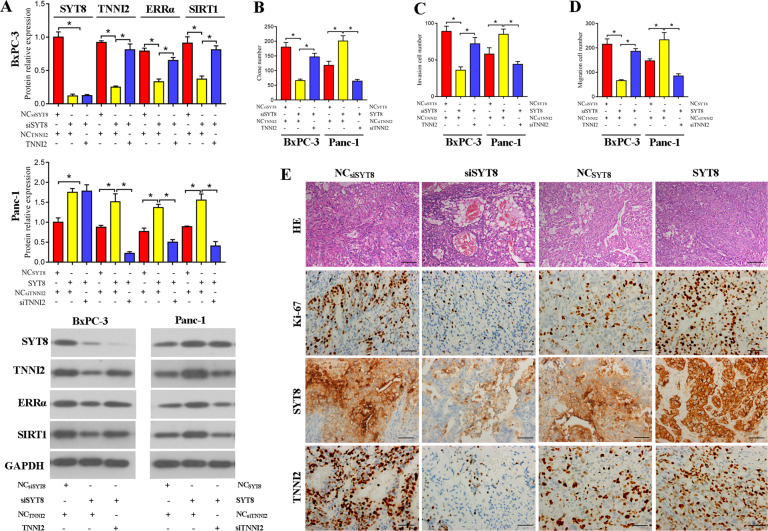
TNNI2 regulates SYT8-induced pancreatic cancer progression via the ERRα/SIRT1 signaling axis. **A** Densitometric quantification and representative western blot images showing SYT8, TNNI2, ERRα, and SIRT1 protein expression levels in BxPC-3 and PANC-1 cells co-expressing different siRNA or overexpression vectors against SYT8 and TNNI2. Glyceraldehyde 3-phosphate dehydrogenase was used as the loading control. **B**–**D** Quantification of cell proliferation from the colony-formation assay. The Transwell assay was used to measure cell migration and invasion in the respective samples. **E** In vivo tumor assay. Representative images of sections from xenograft tumors from BALB/c nude mice subcutaneously injected with BxPC-3 cells and Panc-1 cells transfected with siSYT8 or the SYT8 overexpression vector. Hematoxylin and eosin and immunohistochemical staining were used to visualize Ki-67, SYT8, and TNNI2 expression. Scale bar: 50 μm. Data are presented as mean ± SD with at least three independent experiments. *P < 0.05.

To further verify whether the TNNI2-mediated regulation of the ERRα/SIRT pathway is also altered by SYT8, we overexpressed TNNI2 in siSYT8-expressing BxPC-3 cells or silenced TNNI2 (siTNNI2) in SYT8-overexpressing PANC-1 cells. Evaluation of SYT8, TNNI2, ERRα, and SIRT1 levels by western blotting showed that TNNI2 overexpression had no effect on SYT8 levels but resulted in an increase in ERRα and SIRT1 expression (Fig. 7A). However, the knockdown of TNNI2 decreased the expression of ERRα and SIRT1 (Fig. 7A). This marked increase or decrease in ERRα and SIRT1 levels upon TNNI2 overexpression or knockdown, respectively, also had a direct effect on cell proliferation, migration, and invasion (Fig. 7B–D). To verify the effect of SYT8-mediated regulation in vivo, mice were subcutaneously injected with SYT8 knockdown or SYT8-overexpressing BxPC-3 or PANC-1 cells, respectively, and the tumors were analyzed after 5 weeks. Hematoxylin and eosin staining of tissue sections, along with immunohistochemistry with antibodies against Ki-67 (a proliferation marker), revealed that the tumors in mice that received siSYT8-expressing cells had reduced proliferation and TNNI2 expression (Fig. 7E). In contrast, mice that received cells overexpressing SYT8 exhibited an aberrant tissue architecture characterized by increased cell proliferation and increased TNNI2 expression (Fig. 7E). Collectively, these results suggested that TNNI2 alters SYT8-mediated cell proliferation and migration via ERRα and SIRT1.

### SYT8 positively correlates with TNNI2, ERRα, and SIRT1 in pancreatic cancer

**Fig. 8:**
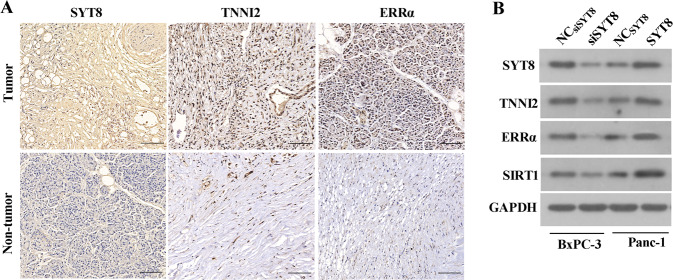
SYT8 positively correlates with TNNI2, ERRα, and SIRT1 in pancreatic cancer. **A** Representative immunohistochemical images depicting the protein expression levels of SYT8, TNNI2, and ERRα in pancreatic cancer tissues and pancreatic non-tumor tissues. Scale bar: 100 μm. B Representative western blot images of TNNI2, ERRα, and SIRT1 protein expression following SYT8 knockdown in the BxPC-3 cell line and SYT8 overexpression in the PANC-1 cell line.

To confirm our in vitro observations in patients, we analyzed healthy tissues and tumors from pancreatic cancer patients. Immunohistochemical analyses of tissue sections showed an increase in TNNI2 and ERRα levels in tumor samples when compared with healthy tissues from patients (Fig. 8A). Additionally, western blot analysis of protein expression in BxPC-3 and PANC-1 cells showed that ERRα, SIRT1, and TNNI2 expressions were downregulated upon SYT8 knockdown in BxPC-3 cells, but upregulated in SYT8-overexpressing PANC-1 cells (Fig. 8B). These results showed a direct positive correlation between SYT8, TNNI2, ERRα, and SIRT1 in vitro and in vivo in patients.

## Discussion

The failure of early diagnosis and its metastatic nature have made pancreatic cancer one of the most lethal cancers, with extremely poor survival [34]. It is therefore important to identify early prognostic markers and gain in-depth knowledge about the mechanisms responsible for the pathogenesis of the disease. In the present study, we characterized the role of the synaptic protein, SYT8, in the progression of pancreatic cancer. We showed that SYT8 was capable of altering the expression levels of key factors that regulate cell metabolism, to promote cell proliferation and invasion in the context of pancreatic cancer. This knowledge may contribute towards the early diagnosis and development of targeted therapy in pancreatic cancer.

Synaptotagmins are a family of transmembrane proteins that are involved in secretory functions, including hormone secretion, vesicle release, and exocytosis [11, 35]. In gastric cancer, SYT8 expression is associated with increased peritoneal metastasis and can potentially serve as a diagnostic and prognostic marker [15]. However, there is no clear understanding of how SYT8 promotes metastasis in pancreatic cancer. We observed an increase in SYT8 expression in pancreatic cancer patient tissues and in vitro cell lines. The expression of SYT8 has previously been shown to be regulated by long-range interaction of the insulin promoter [36]. Constitutive promoter activity results in increased insulin production leading to hyperinsulinemia, a leading cause of pancreatic cancer. Targeting SYT8 using siRNA significantly diminished cell proliferation, migration, and invasion in vitro. However, overexpression of STY8 increased these effects and promoted tumor growth in vivo. These findings were consistent with its previously described role in promoting migration and invasion in gastric cancer cells [15]. In addition to our in vitro and in vivo tumor growth studies, we also performed an in vivo metastasis assay, which further confirmed that SYT8 expression was a significant driver of tumor metastasis as observed in most late-stage pancreatic patients [37].

One of the key known regulators of physiological pathways involved in tumorigenesis, diabetes, and inflammatory diseases is SIRT1, a histone deacetylase that alters metabolic gene expression profiles [21, 38]. Although induction of SIRT1 expression has been shown to induce cell proliferation, migration, and invasion in various pancreatic cancer cell lines [39], the role of SIRT1 in tumorigenesis and cancer progression remains controversial, with several studies suggesting that its role may be tissue-type and context-specific [40]. For example, SIRT1 has been shown to be significantly up-regulated in acute myeloid lymphoma [41], prostate [42] and colon cancer [43], but down-regulated in glioma [44] and gastric cancer [45]. Furthermore, while SIRT1 inhibition has been shown to lead to growth arrest and apoptosis in lymphoma [46] and breast cancer [47], other studies indicate that SIRT1 may act as a tumor suppressor [48, 49]. The role of SIRT1 in pancreatic cancer is complex. Elevated SIRT1 expression has been associated with poorly differentiated pancreatic ductal carcinomas and poor disease outcomes [19] and SIRT1 has been shown to promote pancreatic cancer cell proliferation, invasion and autophagy [50]. In contrast, chemical inhibition or knockdown of SIRT1 in pancreatic cancer cells reduces proliferation and induces apoptosis and senescence [51]. However, SIRT1 inhibition in vivo was found to promote pancreatic cancer xenograft tumor growth [52]. In addition, SIRT1 has been shown to facilitate pancreatic cancer chemoresistance, while application of a combination therapy consisting of a SIRT1 inhibitor and gemcitabine has been shown to have enhanced efficacy for pancreatic cancer [53, 54]. Here, we found that SIRT1 was highly expressed in pancreatic cancer cells. Furthermore, we showed that knockdown of SYT8 also reduced SIRT1 expression. Overexpression of SIRT1 led to a significant increase in the proliferative, migratory and invasive abilities of SYT8 knockdown cells, while SIRT1 knockdown significantly alleviated the effects of SYT8 overexpression. These findings suggest that SYT8 promotes the progression of pancreatic cancer by inducing the expression of SIRT1. However, the mechanism by which SYT8 mediates SIRT1 expression requires further elucidation.

NRs are thought to play a role in the early stages of pancreatic cancer development [55]. The steroid-receptor subfamily of NRs, which includes ERRα, an estrogen receptor-like protein, is involved in the regulation of hormonal imbalance and cellular metabolism [22, 23]. ERRα expression is dysregulated in several types of carcinoma including colon, endometrium, ovary, breast and prostate cancer, and its high expression levels correlate closely with poor patient outcomes in lung adenocarcinoma [56]. Recently, ERRα was shown to be upregulated in pancreatic cancer cell lines, and its expression was directly correlated with promoting cell migration and invasion via the MEK/ERK signaling pathway [57]. Using an ERRα inhibitor, we showed that inhibition of ERRα had a direct effect on SIRT1 expression. Inhibition of ERRα also affected SYT8- and SIRT1-induced cell viability and migration, suggesting that ERRα regulated these processes by altering SIRT1 expression. A previous study has shown that ERRα binds to promoter elements, thereby regulating downstream gene expression [58]. Our immunoprecipitation results indicated interactions of SIRT1 and ERRα at the protein level. Future studies should be conducted to understand ERRα and SIRT1 interaction at a genomic and protein level, to identify the underlying mechanism by which its expression is regulated in pancreatic cancer.

Several NR co-activators, including SRC-1, GRIP1, PNRC, PNRC2, and PGC-1, have been shown to interact with ERRα and enhance its transcriptional activity [59]. However, the interaction between TNNI2 and ERRα in the yeast two-hybrid system is much stronger than that observed between ERRα and these known co-activators. Although troponins such as TNNI2 are generally considered to be involved in the regulation of muscle activity [60, 61], this entire class of proteins has recently been implicated in promoting adenocarcinoma of the lung, pancreas, and stomach [62]. Furthermore, TNNI2 has been identified as a specific biomarker for the prediction of peritoneal metastasis in gastric cancer. However, to date, little is known about the role of TNNI2 and tumorigenesis, and in particular, the association between TNNI2 and pancreatic cancer remains unclear. Interestingly, TNNI2 is a neighboring gene of SYT8, and the insulin promoter has been shown to control the gene expression of both *SYT8* and *TNNI2* in pancreatic islet cells. Thus, we speculated that the TNNI2/ERRα axis was involved in SYT8-mediated pancreatic cancer progression. We showed that TNNI2 promoted pancreatic cancer cell proliferation, migration, and invasion in BxPC-3 and PANC-1 cells. Furthermore, our data indicated that this effect was regulated by SYT8 through the SIRT1/ERRα signaling pathway. Previous studies have reported that ERRα acts as a co-activator of TNNI2 [33]. Thus, it is possible that TTNI2 may also positively regulate its functions in the context of pancreatic cancer. Further studies are required to determine whether other ERRα interactors are also involved in the regulation of SYT8-mediated pancreatic cancer progression.

In conclusion, our study described a fundamental mechanism responsible for the progression of pancreatic cancer, and identified key targets in the SYT8-mediated pathway that were directly related to disease progression, thereby providing the basis for the development of therapeutic targets to reduce the severity of disease and metastasis in patients.

## Materials and methods

### Tissue samples from human subjects

Thirty pairs of pancreatic ductal adenocarcinoma tissues and their matched non-tumor adjacent tissues were obtained from patients who had undergone a pancreatectomy at the Second Affiliated Hospital of Naval Medical University in Shanghai, China. Written informed consent was obtained from all patients, and the study was approved by the Ethical Committee of Second Affiliated Hospital of Naval Medical University, Shanghai, China.

### Cell culture

Four pancreatic cancer cell lines (AsPC-1, BxPC-3, Mia PaCa-2, and Panc-1) and normal HPDE cells were purchased from the Cell Repository, Chinese Academy of Sciences (Shanghai, China). AsPC-1 and BxPC-3 cells were cultured in supplemented RPMI 1640 (Gibco, Gaithersburg, MD, USA) containing 10% fetal bovine serum and antibiotics. Mia PaCa-2 and Panc-1 cells were cultured in fully supplemented Dulbecco Modified Eagle’s Medium (Gibco) as described previously [63]. All cells were maintained at 37 °C/5% CO_2_.

### Cell transfection and treatment

For siRNA-mediated silencing of gene targets, SYT8 siRNA (siSYT8), TNNI2 siRNA (siTNNI2), SIRT1 siRNA (siSIRT1), as well as SYT8, TNNI2, and SIRT1 overexpression vectors were designed and chemically synthesized by Gene Pharma (Shanghai, China). The siRNA vectors were expressed from a lentiviral backbone and transfected into cells seeded in 6-well plates using Lipofectamine RNAi Max (Invitrogen, Carlsbad, CA, USA) according to the manufacturer’s instructions. Cells stably expressing the target siRNA were selected using puromycin for 2 weeks. Sequences of siRNAs used in the study were as follows: siSYT8: 5ʹ-GGCUUAUUCCAGACCUUGU-3ʹ; siTNNI2: 5ʹ-TTGGCATGGGAGATGAGGAGA-3ʹ; and siSIRT1: 5ʹ-GAAGTGCCTCAGATATTAA-3ʹ. For inhibitor experiments, 5 μM of the ERRα inhibitor, C29, (Sigma-Aldrich, St. Louis, MO, USA) was added 24 h prior to harvest.

### Reverse transcription qPCR

Total RNA was isolated from tissues or cells using TRIzol reagent (Invitrogen) according to the manufacturer’s protocol. Reverse transcription was performed using M-MLV Reverse Transcriptase (TaKaRa, Dalian, China) and BulgeLoop™ specific RT-primers (Guangzhou Ribobio, Guangzhou, China). Genes of interest were amplified using gene-specific primers and an SYBR Premix Ex Taq kit (TaKaRa). The following primers were used: *Syt8*, forward: GCTTCTCTCTCCGGTACGTG, reverse: AGGAAGGTGAAGGCCTCATT; *Tnni2*, forward: AGGCAGCACCTGAAGAGTGT, reverse: GTCTTCTGCACCCTCACCTC; *Errα*, forward: CACTATGGTGTGGCATCCTGT, reverse: CGTCTCCGCTTGGTGATCTC; and *Sirt1*, forward: TCATCCTCCATGGGTTCTTC, reverse: TCATCCTCCATGGGTTCTTC.

### Western blotting

For analysis of protein expression, cells were harvested and lysed using RIPA lysis buffer (Sigma-Aldrich) containing freshly added protease inhibitor cocktail (Roche, Basel, Switzerland). The total protein content in cell lysates was evaluated using the Micro BCA Protein Assay Kit (Beyotime, Shanghai, China). A total of 50–100 μg total protein was denatured in sodium dodecyl sulfate-containing sample buffer and loaded onto polyacrylamide gels for separation by electrophoresis (PAGE). Resolved proteins were then blotted onto a polyvinylidene difluoride membrane (Bio-Rad, Hercules, CA, USA) and the membrane was blocked using 5% milk solution [prepared in Tris-buffered saline (TBS)] for 1 h at room temperature. The membranes were then incubated with primary antibodies against SYT8 (LS-C161657; LifeSpan Biosciences, Seattle, WA, USA), TNNI2 (ab184554, Abcam, Cambridge, UK), ERRα (#13826, Cell Signaling Technology, Danvers, MA, USA), or SIRT1 (ab189494, Abcam), and glyceraldehyde 3-phosphate dehydrogenase (#5174, Cell Signaling Technology) prepared in the appropriate dilution in blocking buffer and incubated overnight at 4 °C. After thorough washing, the membranes were incubated with horseradish peroxidase-conjugated secondary antibody (Santa Cruz Biotechnology, Santa Cruz, CA, USA) for 1 h at room temperature. After three washes, the membranes were developed using a substrate (Boster Bio, Pleasanton, CA, USA) and visualized by chemiluminescence.

### Immunoprecipitation

After lysis of cells in RIPA buffer, cell lysates were centrifuged at full speed for 15 min to pellet debris, and the supernatant was incubated with Pierce Protein G Agarose (Pierce, Rockford, IL, USA) and primary antibody or IgG at 4 °C overnight under constant rotation. The beads were thoroughly washed with immunoprecipitation buffer and the immunoprecipitated proteins were eluted from beads in the sample buffer by heating at 100 °C for 5 min. Eluted fractions were subjected to PAGE and western blot analysis.

### Immunohistochemistry

Thin sections of tissue samples were prepared for immunohistochemistry as previously described [63]. Briefly, tissue sections were dewaxed for 20 min in xylene and rehydrated using alcohol. Sections were then incubated with endogenous peroxidase blocking buffer (Beyotime) and treated for antigen retrieval for 20 min at 98 °C. Sections were treated with the primary antibodies used for western blotting overnight at 4 °C, then incubated with corresponding secondary antibodies for 30 min at room temperature. Representative images were chosen based on analysis from more than five different fields of view.

### Cell proliferation and apoptosis assays

To evaluate cell proliferation, 2 × 10^3^ cells were seeded onto 96-well flat-bottom plates and incubated for 0–4 days at 37 °C. At each time point, cells were stained for viability and measured using the Cell Counting Kit-8 assay (Dojindo Laboratories, Kumamoto, Japan) according to the manufacturer’s instructions. Briefly, cells were stained with the CCK-8 reagent provided with the kit and incubated at 37 °C for 2 h prior to measuring the absorbance at 450 nm.

For measuring apoptosis, cells were harvested at the required time points and stained for Annexin V using an Annexin V/PI apoptosis kit (MultiSciences, Hangzhou, China) according to the manufacturer’s instructions. Annexin V-positive cells were analyzed by flow cytometry (BD Biosciences, San Jose, CA, USA).

### Colony-formation assay

Treated PANC-1 and Bxpc-3 cells were harvested and reseeded at a concentration of 500 cells/well in 6-well plates. The cells were cultured for 2 weeks and stained with 0.05% Crystal Violet. The total number of colonies was counted using light microscopy from more than five different fields of view.

### Cell migration and invasion assays

PANC-1 and Bxpc-3 cell migration was assessed using Transwell chambers of 8 μm pore size (BD Biosciences). At 48 h post-treatment, 5 × 10^4^ cells resuspended in 100 μL serum-free medium were seeded into the upper chamber of a Transwell insert. The lower chamber was filled with 700 μL supplemented medium containing 10% fetal bovine serum. The cells were allowed to migrate for 48 h at 37 °C. After incubation, the upper chamber was removed and cells in the lower chamber were washed and stained with 0.05% Crystal Violet. The percentage of migration was evaluated by counting the number of cells in different fields of view.

### Animal experiments

BALB/c male nude mice (4–5 weeks of age, 18–20 g) were purchased from Vitalriver (Beijing, China). BxPC-3 cells stably expressing siRNA were resuspended in 100 μL sterile phosphate-buffered saline and transferred into mice by injection into the right and left dorsal flanks. The mice were raised under pathogen-free conditions and monitored for tumor growth every week. At 5 weeks post-adoptive transfer of cells, the mice were euthanized and the tumors were excised for analysis of tumor volume and then sectioned for immunohistochemistry analysis. The tumor volume was calculated using the formula: volume = (length × width^2^)/2. Six mice are randomly assigned to each group. All animal experimentation was conducted after approval of experimental protocols by the Ethics Committee of Second Affiliated Hospital of Naval Medical University (Shanghai, China).

### In vivo tumorigenicity assay

A total of 2 × 10^6^ treated Panc-1 cells were subcutaneously injected into the right armpit of 6-week-old BALB/c nude mice, and the tumor volume was monitored every week for up to 7 weeks. Tumor volume (mm^3^) was calculated as follows: (shortest diameter)^2^ × (longest diameter)/2.

### In vivo metastasis assay

To examine metastasis in vivo, 1 × 10^6^ luciferase-tagged Panc-1 cells were intravenously injected into the tail vein of BALB/c nude mice. The mice were monitored every week for tumor growth. To assess metastasis, the mice were intraperitoneally injected with 100 μL luciferin, anesthetized with isoflurane, and mice were mounted on an imaging plate to visualize tumor growth by bioluminescence imaging. The mice were imaged using the LuminaXRMS imaging system and analyzed using the Living Image4.3 software (PerkinElmer, Waltham, MA, USA; Fudan University, Shanghai, China). Metastasis was assessed from the images by quantifying the signal intensity, which was representative of the total photons accumulated in a particular area. The mice were euthanized after 7 weeks to prevent adverse events.

### Statistical analysis

All statistical analyses were performed using the SPSS statistical software for Windows, version 17.0 (IBM, Armonk, NY, USA) and presented as an average of biological replicates (mean ± SD). Student’s *t* test or one-way analysis of variance was used to evaluate the differences. All graphs were generated using the GraphPad Prism 6.0 software (GraphPad Software, La Jolla, CA, USA). *P* < 0.05 was considered statistically significant.

## Supplementary information

Detailed Attribution of Authorship

## Data Availability

The datasets used and/or analyzed during the current study are available from the corresponding author on reasonable request.
