# Supplementary material for: SYT8 promotes pancreatic cancer progression via the TNNI2/ERRα/SIRT1 signaling pathway
Source: Cell Death Discov. 2021 Dec 14;7:390. doi: 10.1038/s41420-021-00779-4 (PMC8671424; doi:10.1038/s41420-021-00779-4)
Supplement: Supplementary file 1 — Detailed Attribution of Authorship [file 41420_2021_779_MOESM1_ESM.pdf]

DECLARATION OF CONTRIBUTIONS TO ARTICLE

ADMC

Manuscript Number: CDDISCOVERY--16

Journal Name: *Cell Death Discovery* (the 'Journal')

Proposed Title of the Contribution:

SYT8 promotes pancreatic cancer progression via the TNNI2/ERRα/SIRT1 signaling pathway (the 'Contribution)

Author(s):

Zhiping Fu*, Xing Liang*, Ligang Shi, Liang Tang, Danlei Chen, Anan Liu, Chenghao Shao (the 'Authors')

For all *CDDis*covery articles, each person named as an author in the published version must be able to show he or she has contributed substantially to the article.

Authorship credit should be based on 1) substantial contributions to conception and design, acquisition of data, or analysis and interpretation of data; 2) drafting the article or revising it critically for important intellectual content; and 3) final approval of the version to be published. Authors should meet conditions 1, 2 and 3.

Any person who cannot be shown to have made a substantial contribution to the article cannot be listed as an author in the final version. The name of any person who is deemed to have made a minor contribution can, however, appear in the Acknowledgments section of the article.

Please complete the table below to indicate the contributions of all named authors to the manuscript.

Author Full Name:	Specification of Contribution to the Manuscript:
Zhiping Fu	Substaintial contributions to conception and design the manuscript, experiment executio n and data collection, manuscript preparation and final approval of manuscript
Xing Liang	Experiment execution and data collection, data analysis and interpret ation of data, final manuscript improvement
Ligang Shi	Experiment execution and data collection, manuscript preparation and the final manuscript improvement
Liang Tang	Experiment execution and data collection, data analysis and interpretation of data, manuscript preparation and the final manuscript improvement
Danlei Chen	Experiment execution and data collection, data analysis and interpret ation of data
Anan Liu	Experiment execution and data collection, manuscript preparation and the final manuscript improvement
Chenghao Shao	Substaintial contributions to conception and design the manuscript, manuscrip t preparation and final approval of manuscript

Please complete the table below to indicate the contributions of all named authors to the figures.

Figure 1:

Acquisition of data: Xing Liang, Ligang Shi; Analysis of data: Xing Liang, Liang Tang; Interpretation of data: Xing Liang, Danlei Chen; Assembled the figure: Zhiping Fu

Figure 2:

Acquisition of data: Xing Liang, Ligang Shi; Analysis of data: Liang Tang, Danlei Chen; Interpretation of data: Liang Tang, Danlei Chen; Assembled the figure: Zhiping Fu

Figure 3:

Acquisition of data: Ligang Shi, Anan Liu; Analysis of data: Liang Tang, Danlei Chen; Interpretation of data: Liang Tang, Danlei Chen; Assembled the figure: Zhiping Fu

Figure 4:

Acquisition of data: Zhiping Fu, Xing Liang; Analysis of data: Zhiping Fu, Danlei Chen; Interpretation of data: Liang Tang, Danlei Chen; Assembled the figure: Xing Liang

Figure 5:

Acquisition of data: Xing Liang, Ligang Shi; Analysis of data: Zhiping Fu, Danlei Chen; Interpretation of data: Liang Tang, Danlei Chen; Assembled the figure: Zhiping Fu

Figure 6:

Acquisition of data: Xing Liang, Anan Liu; Analysis of data: Xing Liang, Danlei Chen; Interpretation of data: Liang Tang, Danlei Chen; Assembled the figure: Zhiping Fu

Signed for and on behalf of the Author(s):	Print Name:	Date:
Chenghao Shao	Chenghao Shao	2021.10.21
